# Bis(1,3-dimethyl-1*H*-imidazolium) hexa­fluoro­silicate methanol 0.33-solvate

**DOI:** 10.1107/S1600536813018230

**Published:** 2013-07-06

**Authors:** Chong Tian, Wanli Nie, Maxim V. Borzov

**Affiliations:** a College of Chemistry, Leshan Normal University, Binhe Rd 778, Leshan 614000, Sichuan Province, People’s Republic of China

## Abstract

The title compound, 6C_5_H_9_N_2_^+^·3SiF_6_^2−^·CH_3_OH, (I), was prepared by recrystallization of the crude salt from methanol along with solvent-free 2C_5_H_9_N_2_^+^·SiF_6_^2−^ (II). Crystals of these solvatomorphs can be separated manually. The solvate (I) crystallizes in a rare hexa­gonal space group *P*6/*mcc*. Its asymmetric unit comprises one half of an imidazolium cation bis­ected by the crystallographic *m*-plane, one-sixth and one-twelfth of two crystallographically independent SiF_6_^2–^ dianions (Si atoms are located on the 3.2 and 6/*m* inversion centres), and one-twelfth of a methanol mol­ecule (C atoms are situated on the 622 inversion centres, other atoms are disordered between general positions). In (I), all F atoms of 3.2-located SiF_6_^2–^ dianions participate in the formation of symmetry-equivalent contacts to the H atoms of imidazolium fragments, thus forming rod-type ensembles positioned on the -6 axes. These ‘pillar’ rods are, in turn, F⋯H inter­linked through SiF_6_^2–^ dianions disordered around the 6/*m* centres. The twelvefold disordered methanol mol­ecules are appended to this array by O—H⋯F hydrogen bonds to the 6/*m* located SiF_6_^2–^ dianions. In terms of graph-set notation, the first and second level networks in (I) are *N*_1_ = *C*_2_^2^(7)[3*R*_4_^4^(14)]*D*_2_^2^(4) and *N*_2_ = *D*_2_^2^(5) (C—H⋯O hydrogen bonds are not considered). After locating all symmetrically independent atoms in the cation and anions, there remained a strong (> 3 e Å^−3^) residual electron density peak located at the 622 inversion centre. Treatment of this pre-refined model with the SQUEEZE procedure in *PLATON* [Spek (2009). *Acta Cryst.* D**65**, 148–155] revealed two voids per unit cell, indicative of the presence of the solvent methanol mol­ecule disordered about the 622 inversion centre.

## Related literature

For solvatomorphs of (I), see: Light *et al.* (2007 ▶); Tian *et al.* (2013 ▶). For solvatomorphism of (1,3-dimethyl-1*H*-imidazolium) hexa­fluoro­phosphate, C_5_H_9_N_2_^+^·PF_6_^−^, see: Holbrey *et al.* (2003 ▶). For the practical utility of sterically non-hindered 1,3-dialkyl-1*H*-imidazolium salts with BF_4_^−^ and PF_6_^−^ anions for the preparation of Arduengo carbene adducts with BF_3_ and PF_5_, see: Tian *et al.* (2012 ▶). For graph-set notation, see: Etter *et al.* (1990 ▶); Bernstein *et al.* (1995 ▶); Grell *et al.* (1999 ▶). For a description of the Cambridge Structural Database, see: Allen (2002 ▶). For the SQUEEZE procedure in *PLATON*, see: Spek (2009 ▶).
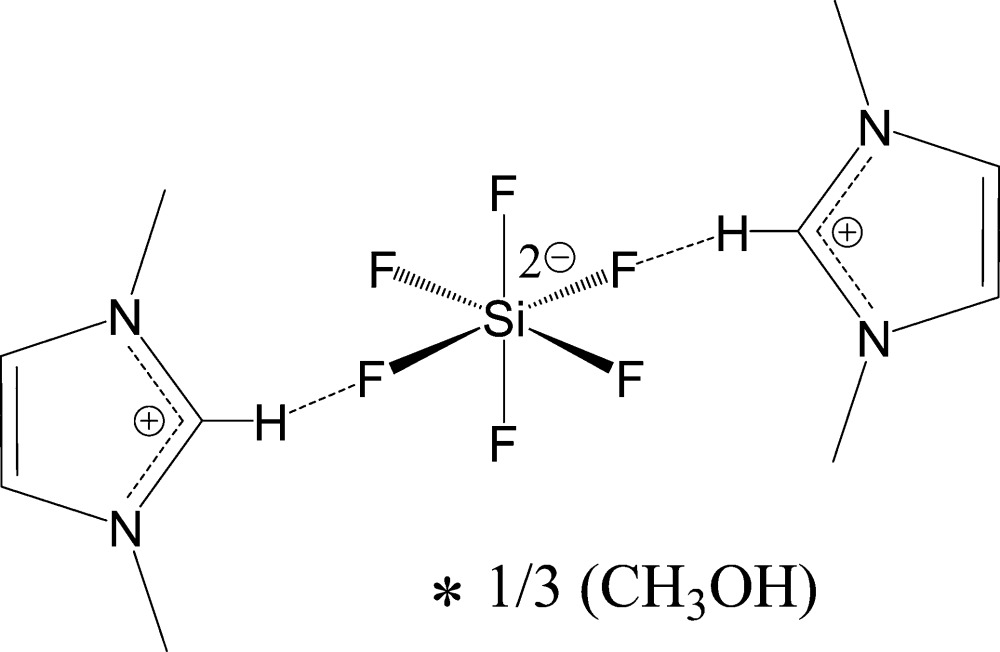


## Experimental

### 

#### Crystal data

6C_5_H_9_N_2_^+^·3SiF_6_^2−^·CH_4_O
*M* *_r_* = 1041.10
Hexagonal, 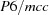

*a* = 12.6577 (7) Å
*c* = 16.8174 (18) Å
*V* = 2333.5 (3) Å^3^
*Z* = 2
Mo *K*α radiation
μ = 0.22 mm^−1^
*T* = 296 K
0.32 × 0.20 × 0.15 mm

#### Data collection

Bruker SMART APEXII diffractometer
Absorption correction: multi-scan (*SADABS*; Sheldrick, 1996 ▶) *T*_min_ = 0.934, *T*_max_ = 0.968
11115 measured reflections
804 independent reflections
623 reflections with *I* > 2σ(*I*)
*R*_int_ = 0.045

#### Refinement

*R*[*F* ^2^ > 2σ(*F* ^2^)] = 0.036
*wR*(*F* ^2^) = 0.098
*S* = 1.10
804 reflections
75 parameters
H atoms treated by a mixture of independent and constrained refinement
Δρ_max_ = 0.15 e Å^−3^
Δρ_min_ = −0.41 e Å^−3^

### 

Data collection: *APEX2* (Bruker, 2007 ▶); cell refinement: *SAINT* (Bruker, 2007 ▶); data reduction: *SAINT*; program(s) used to solve structure: *SHELXS97* (Sheldrick, 2008 ▶); program(s) used to refine structure: *SHELXL97* (Sheldrick, 2008 ▶); molecular graphics: *SHELXTL* (Sheldrick, 2008 ▶) and *OLEX2* (Dolomanov *et al.*, 2009 ▶); software used to prepare material for publication: *SHELXTL* and *OLEX2*.

## Supplementary Material

Crystal structure: contains datablock(s) I, New_Global_Publ_Block. DOI: 10.1107/S1600536813018230/im2435sup1.cif

Structure factors: contains datablock(s) I. DOI: 10.1107/S1600536813018230/im2435Isup2.hkl

Supplementary material file. DOI: 10.1107/S1600536813018230/im2435Isup3.cdx

Additional supplementary materials:  crystallographic information; 3D view; checkCIF report

## Figures and Tables

**Table 1:** Hydrogen-bond geometry (Å, °)

*D*—H⋯*A*	*D*—H	H⋯*A*	*D*⋯*A*	*D*—H⋯*A*
O1—H1*A*⋯F1	0.85	1.96 (1)	2.80 (3)	177 (13)
C1—H1⋯F1	0.94 (3)	2.24 (3)	3.044 (3)	143 (2)
C2—H2⋯F2^i^	0.92 (2)	2.21 (2)	3.095 (2)	160.3 (17)

Symmetry code: (i) 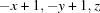.

**Fig. 1.**
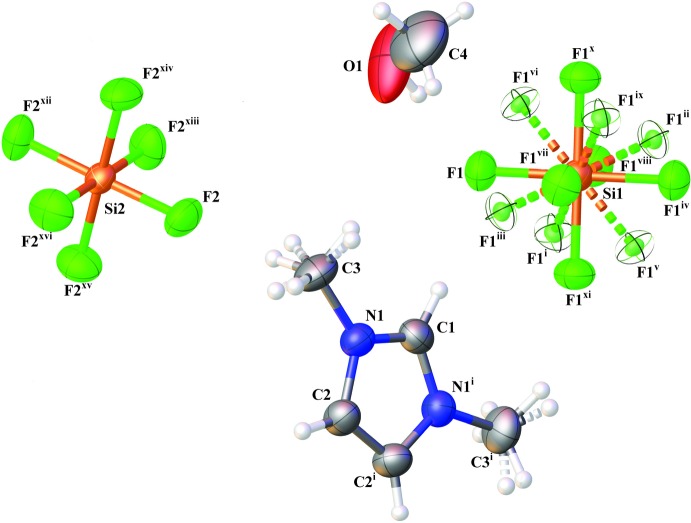
Asymmetric unit of (I) along with equivalent atoms with labelling and thermal ellipsoids at the 50% probability level. F1i-iii,v,vi,ix-atoms (sof 1/2) correspond to the second component of the disordered dianion and are depicted as small spheres surrounded with thermal ellipsoids as rims. Symmetry equivalents of the methanol molecule (disordered between 12 positions) are not depicted for clarity. Symmetry codes: (i) x, y, –z; (ii) –x + y, –x, –z; (iii) x–y, x, z; (iv) –x, –y, –z; (v) –y, x–y, –z; (vi) y, –x + y, z; (vii) –y, x–y, z; (viii) y, –x + y, –z; (ix) –x, –y, z; (x) –x + y, –x, z; (xi) x–y, x, –z; (xii) –x + y+1, y, –z + 1/2; (xiii) –y + 1, x–y, z; (xiv) x, x–y, –z + 1/2; (xv) –x + y+1, –x + 1, z; (xvi) –y + 1, –x + 1, –z + 1/2.

**Fig. 2.**
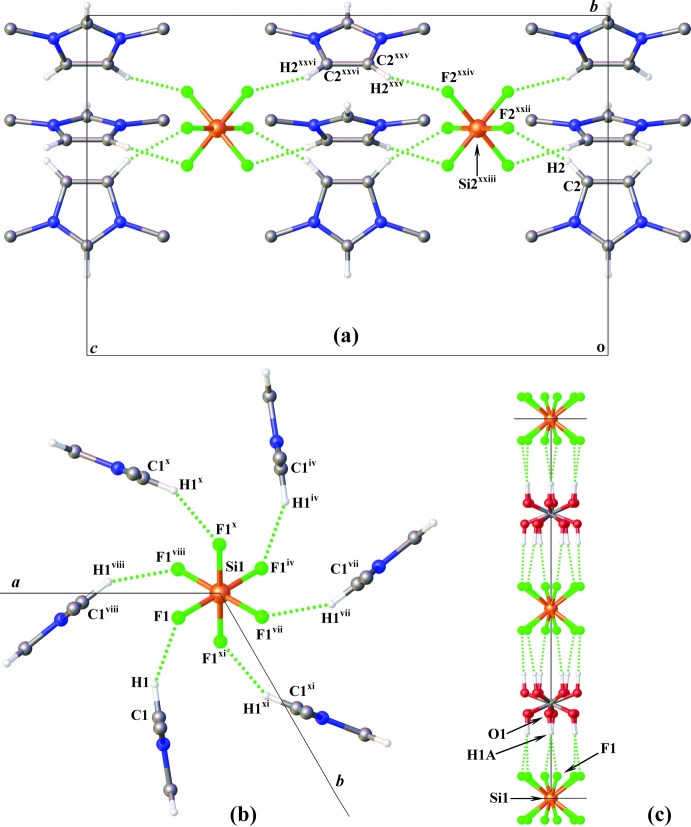
Different motives of C—H···F contacts and O—H···F H-bonds in (I) with labeling. H-atoms of methyl groups are omitted for clarity. C—H···F contacts and H-bonds are depicted as dotted lines. Symmetry codes: (iv) –x, –y, –z; (vii) –y, x–y, z; (viii) y, –x + y, –z; (x) –x + y, –x, z; (xi) x–y, x, –z; (xxii) –x + 1, –y + 1, z; (xxiii) x–y, x, z; (xxiv) –x + 1, –x + y+1, –z + 1/2; (xxv) x, x–y + 1, –z–1/2; (xxvi) x, x–y + 1, z–1/2. (a) The rod-type assembling of the imidazolium cations and the [SiF62–] dianions located at the 3.2 inversion centres. A view along the a-axis. H···F contacts motif C22(7)[3R44(14)]. (b) Linking of the rod ensembles by the [SiF62–] dianions located at the 6/m inversion centres. A view along the c-axis. Methanol molecules are omitted for clarity. Only one of two disordered [SiF62–] dianions is depicted. All shown imidazolium cations belong to different rod-type aggregates. H···F contacts motif D22(4). (c) H-bonding between the twofold disordered [SiF62–] dianions located at the 6/m inversion centres and the 12-fold disordered methanol molecules. A view along the (3, 1, 0) direction. Only crystallographically independent atoms are labeled. H-bonds motif either D22(4) or D.

**Fig. 3.**
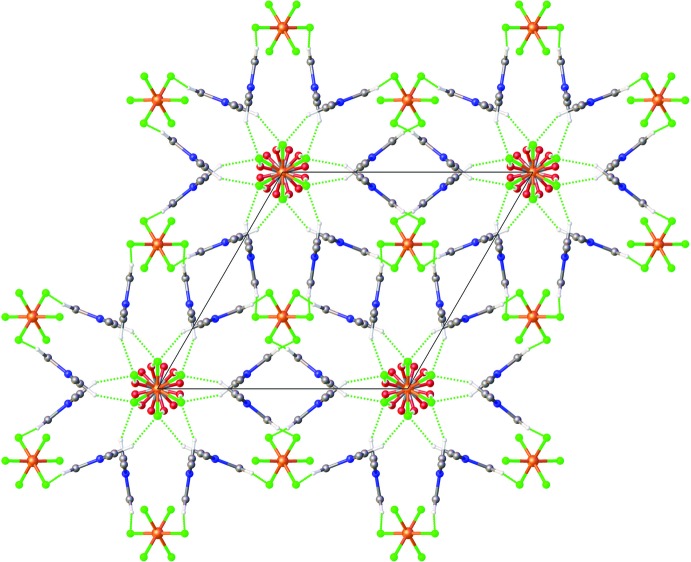
Packing diagram for (II) viewed along the c-axis. H-atoms of methyl groups are omitted for clarity. H···F contacts are depicted as dotted lines. O—H···F H-bonds are eclipsed. The first and second level networks: N1=C22(7)[3R44(14)]D22(4) and N2=D22(5) (C—H···O H-bonds are not considered).

## supplementary crystallographic information

### Comment

Thermolytic decomposition of sterically non-hindered 1,3-dialkyl-1*H*-imidazolium salts with [BF_4_^-^] and [PF_6_^-^] anions present an excellent direct route to Arduengo carbene adducts with BF_3_ and PF_5_ (Tian *et al.*, 2012). Being interested in expanding this reaction for the case of [SiF_6_^2-^], we analysed materials gained by re-crystallization of crude bis(1,3-dimethyl-1*H*-imidazolium) hexafluorosilicate, [C_5_H_9_N_2_^+^]_2_[SiF_6_^2–^], from either methanol or ethanol media. The material obtained from methanol presented both {[C_5_H_9_N_2_^+^]_2_[SiF_6_^2–^]}_3_(CH_3_OH), (I), crystals of which grew at the bottom of a vessel under the layer of the mother liquor and the solvent-free salt, [C_5_H_9_N_2_^+^]_2_[SiF_6_^2–^], (II) (crystals grew on the walls of a vessel above the solution surface during its gradual evaporation into air). Single crystals of (I) and (II) could be easily separated manually. Interestingly, crystallization from ethanol afforded only the solvent-free (II). Identity of the single crystals of (I) prepared from EtOH and MeOH was proved by the unit cell measurements. Details of the structural investigation of (II) can be found in a parallel publication (Tian *et al.*, 2013).

Adduct (I), crystallizes in a rare hexagonal space group *P*6/*mcc*. Analysis of the Cambridge Structural Database [CSD; version 5.32, release May 2013 (Allen, 2002)] reveals only 75 entries and 68 different compounds related to this space group. The asymmetric unit of (I) is depicted in Fig. 1. It comprises one half of the cationic moiety, a one-sixth of a [SiF_6_^2–^] dianion (Si2 atom at the 3.2 inversion centre), a one-twelfth of another [SiF_6_^2–^] dianion (Si1 atom at the 6/*m* inversion centre; the F1 atom site occupancy factor 1/2), and a solvent methanol molecule (C-atom at the 622 inversion centre, each of the other atoms is disordered over 12 general positions). A unit cell of (I) contains 12 [C_5_H_9_N_2_^+^], 6 [SiF_6_^2–^], and 2 CH_3_OH moieties. The cation [C_5_H_9_N_2_^+^] adopts *C*_2_*_v_* point group symmetry and is bisected with the *m*-plane of the crystal lattice. The methyl group of the cation exhibits a slight disorder between two positions [the site occupancy factor of the minor component 0.16 (3)].

In crystal lattice of (I), O—H···F hydrogen bonds and an extended three-dimensional-network of C—H···F contacts are both present (see Table 1). All F-atoms of a 3.2-located [SiF_6_^2–^] dianion participate in C2—H2···F2^xxii^ and symmetry-related contacts [symmetry code: (xxii) –*x* + 1, –*y* + 1, *z*]. These contacts are responsible for formation of rod-type ensembles positioned on the 6 axes which present the "pillar" elements of the entire lattice (see Fig. 2a). In their turn, these rods are F···H interlinked through [SiF_6_^2–^] dianions disordered around the 6/*m* centres. Each 6/*m* located dianion links six different rod-type structures (see Fig. 2 b). The vacancies at the 622 inversion centres are occupied by twelve-fold disordered methanol molecules which are appended to the entire array by O1—H1A···F1 H-bonds (see Fig. 2c). The entire packing diagram is provided in Fig. 3.

Among the closest analogues of (I) and (II), solvatomorphism was observed for (1,3-dimethyl-1*H*-imidazolium) hexafluorophosphate, [C_5_H_9_N_2_^+^][PF_6_^-^], (III), and its semisolvate with benzene (IV) (Holbrey *et al.*, 2003). In that case, inclusion of the solvent molecule resulted not in an increase, but in a decrease of the crystal symmetry [from *Pbca* for (III) down to *P*2_1_/*c* for (IV)]. Interestingly, measurements of the single-crystal of (III) at different temperatures did not reveal any polymorphs (Holbrey *et al.*, 2003).

Crystals of (I) exhibit remarkable stability in air. They do not loose methanol even if evacuated at ambient temperature during a prolonged time (296 K, 0.133 Pa, 5 h). However, when gradually heated in air in a microscopic melting point apparatus, crystals of (I) behave themselves unlike crystals of solvent-free (II) do [m.p. 550 K for (II); Tian *et al.* (2013)]. Within the temperature range of 463–466 K, crystals of (I) start to break down to a powder material which further melts at the same temperature as compound (II) does. These empirical observations explicitly point out the fact that interconversions between the solvatomorphs (I) and (II) are impossible without complete destruction of their crystal lattices.

### Experimental

Crude 1,3-dimethyl-1*H*-imidazolium hexafluorosilicate was prepared by a reaction of 1,3-dimethyl-1*H*-imidazolium iodide and disilver hexafluorosilicate (molar ratio 2:1) in distilled water. Concentrating of the filtrate till dryness followed by re-crystallization from methanol gave both (I) and (II) in an over-all almost quantitative yield. If ethanol is used for re-crystallization, only crystals of (II) are formed. A single crystal of (I) suitable for X-ray diffraction analysis was picked up directly from the material from the bottom of the crystallization vessel. Identity of the single crystals of (II) grown from EtOH and MeOH was proved by unit cell measurements. Melting point measurements were performed with a Microscopic Melting Point X4 apparatus (Beijing MAISIQI High-Tech Co., Ltd.)

### Refinement

The straightforward solution of the structure of (I) in an actual centrosymmetric space group *P*6/*mcc* suggested by *XPREP* failed. The structure was initially solved in a related chiral space group *P*6*cc* and then transferred to *P*6/*mcc* by a corresponding shift along the *c*-axis. After locating all symmetrically independent atoms in the cation and anions [C1, H1 (*sof*-s 1/2), C2, H2, N1, C3, H3A—C, Si1 (*sof* 0.08333), F1 (*sof* 0.5; disordered over the 6/*m* centre, assigned to PART -1), Si2 (*sof* 0.16667), and F2 (*sof* 1); all *sof*-s fixed], there still remained a strong (> 3 e Å^-3^) residual electron density peak (Q-peak) located at the 622 inversion centre. Treatment of this pre-refined model with the SQUEEZE procedure of the *PLATON* program package (Spek, 2009) revealed two voids per unit cell at (0, 0, 1/4) and (0, 0, 3/4) (each of 65 Å^3^ and 19 e; total electron count per unit cell 38 e) that was indicative of the presence of the solvent methanol molecule disordered around the 622 inversion centre. Thus, the strongest Q-peak was assigned a C-type (C4; fixed *sof* 0.08333). The next refinement cycle retrieved a weaker Q-peak at a general position approximately 1.2 Å away from C4 which was further treated as an O-atom disordered between 12 symmetry equivalent positions (O1; fixed *sof* 0.08333). A Q-peak nearly located on the 6-fold inversion axis approximately 1.0 Å away from C4 was used as a basis for the calculation of the methanol molecule H-atoms positions (treated further as riding atoms with all *sof*-s fixed to 0.08333). Position of the remaining hydroxyl group H-atom was not evident from the difference Fourier synthesis (a disorder between 12 positions). However, a comparatively short O1···F1 distance [2.80 (3) Å] suggested a presence of an H-bond connecting these two atoms. This way, H-atom (H1A) was put 0.85 Å away from O1 on the line connecting O1 and F1 atoms and treated as a riding atom (AFIX 3 instruction) with *sof* fixed to 0.08333. Disordered O1, H4A—C, and H1A atoms were all assigned to PART -2. At the final step of the refinement, a noticeable disorder of the methyl group in the imidazolium moiety [minor component *sof* 0.16 (3)] was also taken into account. All non-H atoms were refined anisotropically. Atoms H1 and H2 were found from the difference Fourier synthesis and refined isotropically. The methyl groups H atoms were treated as riding atoms with distance C—H = 0.96 and *U*_iso_(H) = 1.5 *U*_eq_(C). Hydroxyl group H-atom was treated as a riding atom with distance O—H = 0.85 and *U*_iso_(H) = 1.2 *U*_eq_(O).

### Crystal data

**Table:**

6C_5_H_9_N_2_^+^·3SiF_6_^2^^−^·CH_4_O	*D*_x_ = 1.482 Mg m^−^^3^
*M**_r_* = 1041.10	Melting point: 550 K
Hexagonal, *P*6/*m**c**c*	Mo *K*α radiation, λ = 0.71073 Å
Hall symbol: -P 6 2c	Cell parameters from 4399 reflections
*a* = 12.6577 (7) Å	θ = 2.4–28.0°
*c* = 16.8174 (18) Å	µ = 0.22 mm^−^^1^
*V* = 2333.5 (3) Å^3^	*T* = 296 K
*Z* = 2	Block, yellow
*F*(000) = 1080	0.32 × 0.20 × 0.15 mm

### Data collection

**Table:**

Bruker SMART APEXII diffractometer	804 independent reflections
Radiation source: fine-focus sealed tube	623 reflections with *I* > 2σ(*I*)
Graphite monochromator	*R*_int_ = 0.045
Detector resolution: 8.333 pixels mm^-1^	θ_max_ = 26.0°, θ_min_ = 1.9°
phi and ω scans	*h* = −15→11
Absorption correction: multi-scan (*SADABS*; Sheldrick, 1996)	*k* = −12→15
*T*_min_ = 0.934, *T*_max_ = 0.968	*l* = −20→20
11115 measured reflections	

### Refinement

**Table:**

Refinement on *F*^2^	Primary atom site location: structure-invariant direct methods
Least-squares matrix: full	Secondary atom site location: difference Fourier map
*R*[*F*^2^ > 2σ(*F*^2^)] = 0.036	Hydrogen site location: inferred from neighbouring sites
*wR*(*F*^2^) = 0.098	H atoms treated by a mixture of independent and constrained refinement
*S* = 1.10	*w* = 1/[σ^2^(*F*_o_^2^) + (0.056*P*)^2^ + 0.3029*P*] where *P* = (*F*_o_^2^ + 2*F*_c_^2^)/3
804 reflections	(Δ/σ)_max_ < 0.001
75 parameters	Δρ_max_ = 0.15 e Å^−^^3^
0 restraints	Δρ_min_ = −0.41 e Å^−^^3^

### Special details

**Table:**

Geometry. All e.s.d.'s (except the e.s.d. in the dihedral angle between two l.s. planes) are estimated using the full covariance matrix. The cell e.s.d.'s are taken into account individually in the estimation of e.s.d.'s in distances, angles and torsion angles; correlations between e.s.d.'s in cell parameters are only used when they are defined by crystal symmetry. An approximate (isotropic) treatment of cell e.s.d.'s is used for estimating e.s.d.'s involving l.s. planes.
Refinement. Refinement of *F*^2^ against ALL reflections. The weighted *R*-factor *wR* and goodness of fit *S* are based on *F*^2^, conventional *R*-factors *R* are based on *F*, with *F* set to zero for negative *F*^2^. The threshold expression of *F*^2^ > σ(*F*^2^) is used only for calculating *R*-factors(gt) *etc*. and is not relevant to the choice of reflections for refinement. *R*-factors based on *F*^2^ are statistically about twice as large as those based on *F*, and *R*- factors based on ALL data will be even larger.

### Fractional atomic coordinates and isotropic or equivalent isotropic displacement parameters (Å^2^)

**Table:**

	*x*	*y*	*z*	*U*_iso_*/*U*_eq_	Occ. (<1)
N1	0.32338 (12)	0.39168 (13)	0.06402 (9)	0.0388 (4)	
C1	0.2983 (2)	0.3220 (2)	0.0000	0.0404 (6)	
H1	0.266 (2)	0.237 (3)	0.0000	0.051 (7)*	
C2	0.36659 (17)	0.51001 (18)	0.03979 (11)	0.0430 (4)	
H2	0.389 (2)	0.571 (2)	0.0769 (12)	0.056 (6)*	
C3	0.3094 (2)	0.3496 (2)	0.14668 (13)	0.0560 (6)	
H3BD	0.2418	0.2678	0.1504	0.084*	0.84 (3)
H3BE	0.2946	0.4023	0.1801	0.084*	0.84 (3)
H3BF	0.3825	0.3511	0.1636	0.084*	0.84 (3)
H3AA	0.2256	0.2899	0.1564	0.084*	0.16 (3)
H3AB	0.3337	0.4176	0.1819	0.084*	0.16 (3)
H3AC	0.3597	0.3139	0.1558	0.084*	0.16 (3)
Si1	0.0000	0.0000	0.0000	0.0319 (4)	
F1	0.12523 (16)	0.0637 (2)	0.05780 (10)	0.0459 (5)	0.50
Si2	0.6667	0.3333	0.2500	0.0318 (3)	
F2	0.55741 (10)	0.33189 (10)	0.19222 (6)	0.0525 (4)	
C4	0.0000	0.0000	0.2500	0.115 (4)	
H4A	0.0030	0.0004	0.3070	0.138*	0.0833333
H4B	−0.0606	−0.0794	0.2318	0.138*	0.0833333
H4C	−0.0207	0.0595	0.2322	0.138*	0.0833333
O1	0.102 (5)	0.025 (17)	0.2226 (9)	0.115 (19)	0.0833333
H1A	0.1086	0.0345	0.1725	0.138*	0.0833333

### Atomic displacement parameters (Å^2^)

**Table:**

	*U*^11^	*U*^22^	*U*^33^	*U*^12^	*U*^13^	*U*^23^
N1	0.0411 (8)	0.0412 (8)	0.0345 (8)	0.0209 (7)	0.0001 (6)	0.0030 (6)
C1	0.0377 (13)	0.0359 (14)	0.0443 (15)	0.0159 (11)	0.000	0.000
C2	0.0544 (11)	0.0407 (10)	0.0386 (9)	0.0273 (9)	−0.0032 (8)	−0.0029 (8)
C3	0.0683 (13)	0.0664 (13)	0.0373 (10)	0.0367 (11)	0.0040 (9)	0.0137 (9)
Si1	0.0316 (5)	0.0316 (5)	0.0324 (8)	0.0158 (3)	0.000	0.000
F1	0.0390 (10)	0.0499 (13)	0.0438 (11)	0.0184 (11)	−0.0091 (9)	−0.0027 (10)
Si2	0.0362 (4)	0.0362 (4)	0.0230 (5)	0.0181 (2)	0.000	0.000
F2	0.0504 (7)	0.0644 (7)	0.0466 (7)	0.0316 (6)	−0.0081 (5)	0.0106 (5)
C4	0.145 (7)	0.145 (7)	0.056 (7)	0.072 (4)	0.000	0.000
O1	0.08 (2)	0.21 (5)	0.048 (10)	0.07 (4)	−0.005 (19)	−0.04 (5)

### Geometric parameters (Å, º)

**Table:**

N1—C1	1.326 (2)	C3—H3AA	0.9600
N1—C2	1.375 (2)	C3—H3AB	0.9600
N1—C3	1.467 (2)	C3—H3AC	0.9600
C1—N1^i^	1.326 (2)	Si1—F1	1.6821 (17)
C1—H1	0.94 (3)	Si2—F2	1.6829 (10)
C2—C2^i^	1.338 (4)	C4—O1	1.25 (2)
C2—H2	0.92 (2)	C4—H4A	0.9600
C3—H3BD	0.9600	C4—H4B	0.9600
C3—H3BE	0.9600	C4—H4C	0.9600
C3—H3BF	0.9600	O1—H1A	0.8498
			
C1—N1—C2	108.46 (16)	O1^x^—C4—O1^ix^	55.4 (3)
C1—N1—C3	125.63 (17)	O1^xiv^—C4—O1^xvii^	55.4 (3)
C2—N1—C3	125.90 (16)	O1^x^—C4—O1^xvii^	150 (10)
N1^i^—C1—N1	108.6 (2)	O1^ix^—C4—O1^xvii^	96 (10)
N1^i^—C1—H1	125.69 (12)	O1^xiv^—C4—O1^xviii^	136.8 (15)
N1—C1—H1	125.69 (12)	O1^x^—C4—O1^xviii^	51 (10)
C2^i^—C2—N1	107.25 (10)	O1^ix^—C4—O1^xviii^	53 (10)
C2^i^—C2—H2	132.8 (13)	O1^xvii^—C4—O1^xviii^	107.3 (8)
N1—C2—H2	120.0 (13)	O1^xiv^—C4—O1^iii^	51 (10)
N1—C3—H3BD	109.5	O1^x^—C4—O1^iii^	136.8 (15)
N1—C3—H3BE	109.5	O1^ix^—C4—O1^iii^	107.3 (8)
H3BD—C3—H3BE	109.5	O1^xvii^—C4—O1^iii^	53 (10)
N1—C3—H3BF	109.5	O1^xviii^—C4—O1^iii^	154 (10)
H3BD—C3—H3BF	109.5	O1^xiv^—C4—O1^vi^	100 (10)
H3BE—C3—H3BF	109.5	O1^x^—C4—O1^vi^	55.4 (3)
N1—C3—H3AA	109.5	O1^ix^—C4—O1^vi^	107.3 (8)
N1—C3—H3AB	109.5	O1^xvii^—C4—O1^vi^	154 (10)
H3AA—C3—H3AB	109.5	O1^xviii^—C4—O1^vi^	96 (10)
N1—C3—H3AC	109.5	O1^iii^—C4—O1^vi^	107.3 (8)
H3AA—C3—H3AC	109.5	O1^xiv^—C4—O1^xix^	55.4 (3)
H3AB—C3—H3AC	109.5	O1^x^—C4—O1^xix^	100 (10)
F1^ii^—Si1—F1^iii^	180.00 (19)	O1^ix^—C4—O1^xix^	154 (10)
F1^ii^—Si1—F1^iv^	48.16 (4)	O1^xvii^—C4—O1^xix^	107.3 (8)
F1^iii^—Si1—F1^iv^	131.84 (4)	O1^xviii^—C4—O1^xix^	107.3 (8)
F1^ii^—Si1—F1^v^	89.94 (9)	O1^iii^—C4—O1^xix^	96 (10)
F1^iii^—Si1—F1^v^	90.06 (9)	O1^vi^—C4—O1^xix^	53 (10)
F1^iv^—Si1—F1^v^	48.16 (4)	O1^xiv^—C4—O1^xx^	107.3 (8)
F1^ii^—Si1—F1^vi^	90.06 (9)	O1^x^—C4—O1^xx^	53 (10)
F1^iii^—Si1—F1^vi^	89.94 (9)	O1^ix^—C4—O1^xx^	100 (10)
F1^iv^—Si1—F1^vi^	131.84 (4)	O1^xvii^—C4—O1^xx^	136.8 (15)
F1^v^—Si1—F1^vi^	180.00 (13)	O1^xviii^—C4—O1^xx^	55.4 (3)
F1^ii^—Si1—F1^vii^	131.84 (4)	O1^iii^—C4—O1^xx^	150 (10)
F1^iii^—Si1—F1^vii^	48.16 (4)	O1^vi^—C4—O1^xx^	51 (10)
F1^iv^—Si1—F1^vii^	90.06 (9)	O1^xix^—C4—O1^xx^	55.4 (3)
F1^v^—Si1—F1^vii^	70.61 (12)	O1^xiv^—C4—O1^xxi^	107.3 (8)
F1^vi^—Si1—F1^vii^	109.39 (12)	O1^x^—C4—O1^xxi^	96 (10)
F1^ii^—Si1—F1^viii^	48.16 (4)	O1^ix^—C4—O1^xxi^	51 (10)
F1^iii^—Si1—F1^viii^	131.84 (4)	O1^xvii^—C4—O1^xxi^	55.4 (3)
F1^iv^—Si1—F1^viii^	89.94 (9)	O1^xviii^—C4—O1^xxi^	55.4 (3)
F1^v^—Si1—F1^viii^	109.39 (12)	O1^iii^—C4—O1^xxi^	100 (10)
F1^vi^—Si1—F1^viii^	70.61 (12)	O1^vi^—C4—O1^xxi^	150 (10)
F1^vii^—Si1—F1^viii^	180.00 (13)	O1^xix^—C4—O1^xxi^	136.8 (15)
F1^ii^—Si1—F1^ix^	90.06 (9)	O1^xx^—C4—O1^xxi^	107.3 (8)
F1^iii^—Si1—F1^ix^	89.94 (9)	O1^xiv^—C4—O1^vii^	96 (10)
F1^iv^—Si1—F1^ix^	70.61 (12)	O1^x^—C4—O1^vii^	107.3 (8)
F1^v^—Si1—F1^ix^	90.06 (9)	O1^ix^—C4—O1^vii^	55.4 (3)
F1^vi^—Si1—F1^ix^	89.94 (9)	O1^xvii^—C4—O1^vii^	51 (10)
F1^vii^—Si1—F1^ix^	48.16 (4)	O1^xviii^—C4—O1^vii^	100 (10)
F1^viii^—Si1—F1^ix^	131.84 (4)	O1^iii^—C4—O1^vii^	55.4 (3)
F1^ii^—Si1—F1^i^	89.94 (9)	O1^vi^—C4—O1^vii^	136.8 (15)
F1^iii^—Si1—F1^i^	90.06 (9)	O1^xix^—C4—O1^vii^	150 (10)
F1^iv^—Si1—F1^i^	109.39 (12)	O1^xx^—C4—O1^vii^	154 (10)
F1^v^—Si1—F1^i^	89.94 (9)	O1^xxi^—C4—O1^vii^	53 (10)
F1^vi^—Si1—F1^i^	90.06 (9)	O1^xiv^—C4—O1	53 (10)
F1^vii^—Si1—F1^i^	131.84 (4)	O1^x^—C4—O1	107.3 (8)
F1^viii^—Si1—F1^i^	48.16 (4)	O1^ix^—C4—O1	136.8 (15)
F1^ix^—Si1—F1^i^	180.00 (14)	O1^xvii^—C4—O1	100 (10)
F1^ii^—Si1—F1^x^	70.61 (12)	O1^xviii^—C4—O1	150 (10)
F1^iii^—Si1—F1^x^	109.39 (12)	O1^iii^—C4—O1	55.4 (3)
F1^iv^—Si1—F1^x^	90.06 (9)	O1^vi^—C4—O1	55.4 (3)
F1^v^—Si1—F1^x^	131.84 (4)	O1^xix^—C4—O1	51 (10)
F1^vi^—Si1—F1^x^	48.16 (4)	O1^xx^—C4—O1	96 (10)
F1^vii^—Si1—F1^x^	89.94 (9)	O1^xxi^—C4—O1	154 (10)
F1^viii^—Si1—F1^x^	90.06 (9)	O1^vii^—C4—O1	107.3 (8)
F1^ix^—Si1—F1^x^	48.16 (4)	O1^xiv^—C4—H4A	66.7
F1^i^—Si1—F1^x^	131.84 (4)	O1^x^—C4—H4A	112.4
F1^ii^—Si1—F1^xi^	109.39 (12)	O1^ix^—C4—H4A	113.7
F1^iii^—Si1—F1^xi^	70.61 (12)	O1^xvii^—C4—H4A	68.7
F1^iv^—Si1—F1^xi^	89.94 (9)	O1^xviii^—C4—H4A	70.1
F1^v^—Si1—F1^xi^	48.16 (4)	O1^iii^—C4—H4A	110.7
F1^vi^—Si1—F1^xi^	131.84 (4)	O1^vi^—C4—H4A	110.3
F1^vii^—Si1—F1^xi^	90.06 (9)	O1^xix^—C4—H4A	66.4
F1^viii^—Si1—F1^xi^	89.94 (9)	O1^xx^—C4—H4A	68.1
F1^ix^—Si1—F1^xi^	131.84 (4)	O1^xxi^—C4—H4A	70.4
F1^i^—Si1—F1^xi^	48.16 (4)	O1^vii^—C4—H4A	112.9
F1^x^—Si1—F1^xi^	180.00 (19)	O1—C4—H4A	109.5
F1^ii^—Si1—F1	131.84 (4)	O1^xiv^—C4—H4B	154.5
F1^iii^—Si1—F1	48.16 (4)	O1^x^—C4—H4B	3.1
F1^iv^—Si1—F1	180.0	O1^ix^—C4—H4B	55.4
F1^v^—Si1—F1	131.83 (4)	O1^xvii^—C4—H4B	148.9
F1^vi^—Si1—F1	48.17 (4)	O1^xviii^—C4—H4B	48.1
F1^vii^—Si1—F1	89.94 (9)	O1^iii^—C4—H4B	139.8
F1^viii^—Si1—F1	90.06 (9)	O1^vi^—C4—H4B	56.8
F1^ix^—Si1—F1	109.39 (12)	O1^xix^—C4—H4B	99.4
F1^i^—Si1—F1	70.61 (12)	O1^xx^—C4—H4B	51.6
F1^x^—Si1—F1	89.95 (9)	O1^xxi^—C4—H4B	94.1
F1^xi^—Si1—F1	90.05 (9)	O1^vii^—C4—H4B	108.2
F2^xii^—Si2—F2^xiii^	89.25 (7)	O1—C4—H4B	109.5
F2^xii^—Si2—F2^xiv^	89.99 (6)	H4A—C4—H4B	109.5
F2^xiii^—Si2—F2^xiv^	90.77 (8)	O1^xiv^—C4—H4C	95.1
F2^xii^—Si2—F2^xv^	90.77 (8)	O1^x^—C4—H4C	108.7
F2^xiii^—Si2—F2^xv^	89.99 (6)	O1^ix^—C4—H4C	55.7
F2^xiv^—Si2—F2^xv^	178.93 (8)	O1^xvii^—C4—H4C	48.5
F2^xii^—Si2—F2^xvi^	89.99 (6)	O1^xviii^—C4—H4C	98.1
F2^xiii^—Si2—F2^xvi^	178.93 (8)	O1^iii^—C4—H4C	56.6
F2^xiv^—Si2—F2^xvi^	89.99 (6)	O1^vi^—C4—H4C	140.2
F2^xv^—Si2—F2^xvi^	89.25 (7)	O1^xix^—C4—H4C	150.0
F2^xii^—Si2—F2	178.93 (8)	O1^xx^—C4—H4C	153.2
F2^xiii^—Si2—F2	89.99 (6)	O1^xxi^—C4—H4C	50.5
F2^xiv^—Si2—F2	89.25 (7)	O1^vii^—C4—H4C	3.4
F2^xv^—Si2—F2	89.99 (6)	O1—C4—H4C	109.5
F2^xvi^—Si2—F2	90.77 (8)	H4A—C4—H4C	109.5
O1^xiv^—C4—O1^x^	154 (10)	H4B—C4—H4C	109.5
O1^xiv^—C4—O1^ix^	150 (10)	C4—O1—H1A	114.9
			
C2—N1—C1—N1^i^	−0.3 (3)	C1—N1—C2—C2^i^	0.18 (16)
C3—N1—C1—N1^i^	−179.32 (13)	C3—N1—C2—C2^i^	179.20 (14)

Symmetry codes: (i) *x*, *y*, −*z*; (ii) −*x*+*y*, −*x*, −*z*; (iii) *x*−*y*, *x*, *z*; (iv) −*x*, −*y*, −*z*; (v) −*y*, *x*−*y*, −*z*; (vi) *y*, −*x*+*y*, *z*; (vii) −*y*, *x*−*y*, *z*; (viii) *y*, −*x*+*y*, −*z*; (ix) −*x*, −*y*, *z*; (x) −*x*+*y*, −*x*, *z*; (xi) *x*−*y*, *x*, −*z*; (xii) −*x*+*y*+1, *y*, −*z*+1/2; (xiii) −*y*+1, *x*−*y*, *z*; (xiv) *x*, *x*−*y*, −*z*+1/2; (xv) −*x*+*y*+1, −*x*+1, *z*; (xvi) −*y*+1, −*x*+1, −*z*+1/2; (xvii) *y*, *x*, −*z*+1/2; (xviii) −*x*, −*x*+*y*, −*z*+1/2; (xix) *x*−*y*, −*y*, −*z*+1/2; (xx) −*y*, −*x*, −*z*+1/2; (xxi) −*x*+*y*, *y*, −*z*+1/2.

### Hydrogen-bond geometry (Å, º)

**Table:**

*D*—H···*A*	*D*—H	H···*A*	*D*···*A*	*D*—H···*A*
O1—H1*A*···F1	0.85	1.96 (1)	2.80 (3)	177 (13)
C1—H1···F1	0.94 (3)	2.24 (3)	3.044 (3)	143 (2)
C2—H2···F2^xxii^	0.92 (2)	2.21 (2)	3.095 (2)	160.3 (17)

Symmetry code: (xxii) −*x*+1, −*y*+1, *z*.

### Table 2. C—H···F contacts and O—H···F H-bond geometry (Å, °) in (II).

**Table:**

*D*—H···*A*	*D*—H	H···*A*	*D*···*A*	*D*—H···*A*
O1—H1A···F1	0.85	1.955 (2)	2.80 (3)	177 (13)
C1—H1···F1	0.94 (3)	2.24 (3)	3.044 (3)	143 (2)
C2—H2···F2^xxii^	0.92 (2)	2.21 (2)	3.095 (2)	160 (2)

Symmetry code: (xxii): –*x*+1, –*y*+1, *z*.

## References

[bb1] Allen, F. H. (2002). *Acta Cryst.* B**58**, 380–388. 10.1107/s0108768102003890 12037359

[bb2] Bernstein, J., Davis, R. E., Shimoni, L. & Chang, N.-L. (1995). *Angew. Chem. Int. Ed. Engl.* **34**, 1555–1573.

[bb3] Bruker (2007). *APEX2* and *SAINT* Bruker AXS Inc., Madison, Wisconsin, USA.

[bb4] Dolomanov, O. V., Bourhis, L. J., Gildea, R. J., Howard, J. A. K. & Puschmann, H. (2009). *J. Appl. Cryst.* **42**, 339–341.

[bb5] Etter, M. C., MacDonald, J. C. & Bernstein, J. (1990). *Acta Cryst.* B**46**, 256–262. 10.1107/s0108768189012929 2344397

[bb6] Grell, J., Bernstein, J. & Tinhofer, G. (1999). *Graph Set Analysis of Some Hydrogen Bond Patterns. Some Mathematical Concepts*, edited by H. Wähling. München: Fakultät für Mathematik und Informatik, Technische Universität München.

[bb7] Holbrey, J. D., Reichert, W. M. & Nieuwenhuyzen, M. (2003). *Chem. Commun.* pp. 476–477. 10.1039/b212726a 12638957

[bb8] Light, M. E., Bates, G. W. & Gale, P. A. (2007). Private communication (refcode NIQFAV). CCDC, Cambridge, England.

[bb9] Sheldrick, G. M. (1996). *SADABS* University of Göttingen, Germany.

[bb10] Sheldrick, G. M. (2008). *Acta Cryst.* A**64**, 112–122. 10.1107/S0108767307043930 18156677

[bb11] Spek, A. L. (2009). *Acta Cryst.* D**65**, 148–155. 10.1107/S090744490804362X PMC2631630 19171970

[bb12] Tian, C., Nie, W. & Borzov, M. V. (2013). *Acta Cryst* E**69**, o1218–o1219. 10.1107/S1600536813018242 PMC3793725 24109312

[bb13] Tian, C., Nie, W., Borzov, M. V. & Su, P. (2012). *Organometallics*, **31**, 1751–1760.

